# Iranian neurological events: The 1^st^ Iranian congress of neurointervention

**Published:** 2013

**Authors:** Shirin Jamal Omidi

**Affiliations:** 1 Resident, Department of Neurology, Shariati Hospital, Tehran University of Medical Sciences, Tehran, Iran

**Keywords:** Iran, Congress, Neurointervention

Neurointervention has fast found its way through treatment of cerebrovascular diseases. Being less invasive and associated with less post-operative morbidity than the open surgical methods, it is of growing interest among neurologists and neurosurgeons around the world. Since the introduction of this field in Iran for almost a decade ago, many patients with neurologic problems have benefited from endovascular operations in Iran.

**Figure 1:**
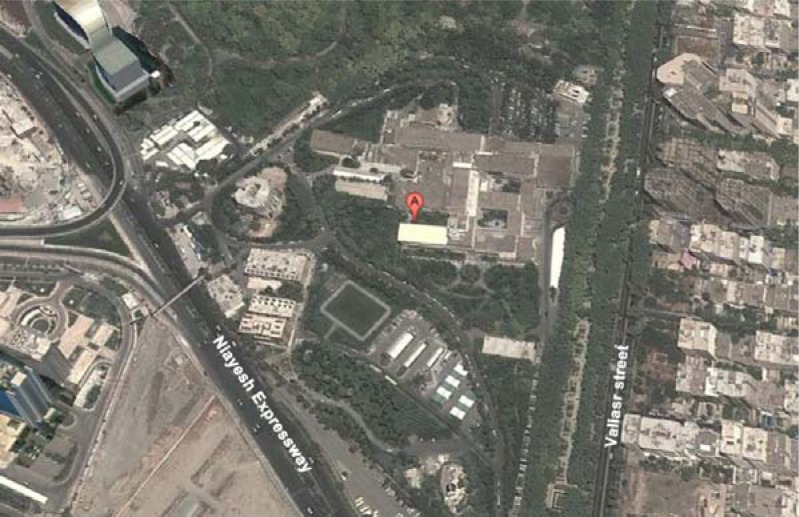
Rajaee Heart Center in Tehran (A) (from Google Earth, accessed 07-09-2013)

Aiming to provide neurologists and neurosurgeons with more information on different aspects of neurointervention, as well as introducing recent developments in endovascular techniques to the related specialists in Iran and the neighboring countries, the first Iranian Congress of Neurointervention was held in Shahid Rajaee Heart Centre, Tehran, Iran (Fig. 1) in 29^th^ and 30^th^ August 2013, supported by the Iranian Association of Neurology and Iranian Association of stroke. Approximately, 150 professionals including neurologists, neurosurgeons, cardiologists and radiologists from across Iran and other countries including Oman, Turkey, Iraq, France, and the U.S. contributed into this event.

**Figure 2:**
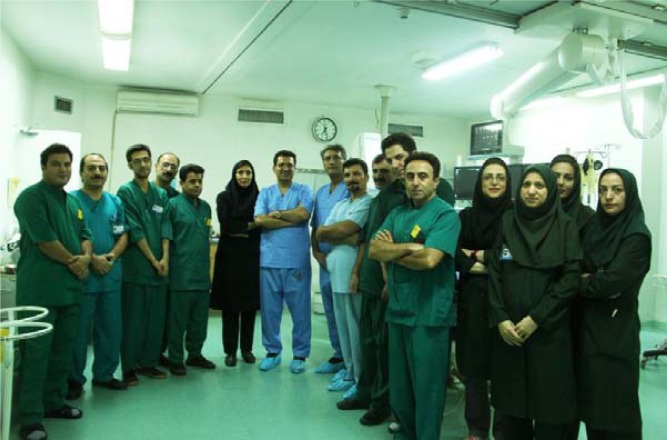
Neurointerventional team including neurologists, angiography nurses and technicians

**Figure 3:**
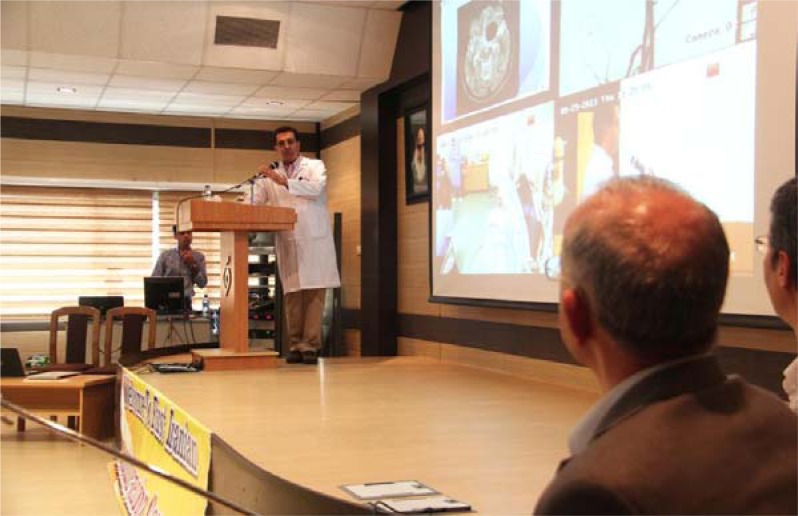
Nenurointerventional panel with concurrent live aneurysm coiling

**Figure 4:**
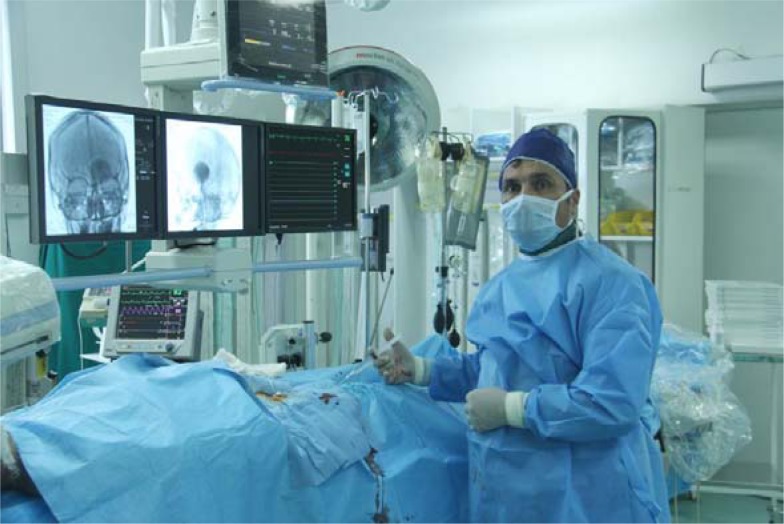
Neurointerventionalist in catheterization laboratory during giant aneurysm coiling

**Figure 5:**
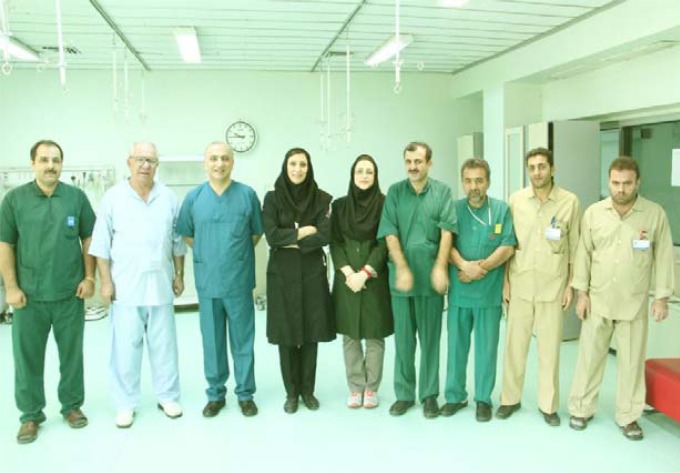
Interventional cardiology team before carotid angioplasty

**Figure 6:**
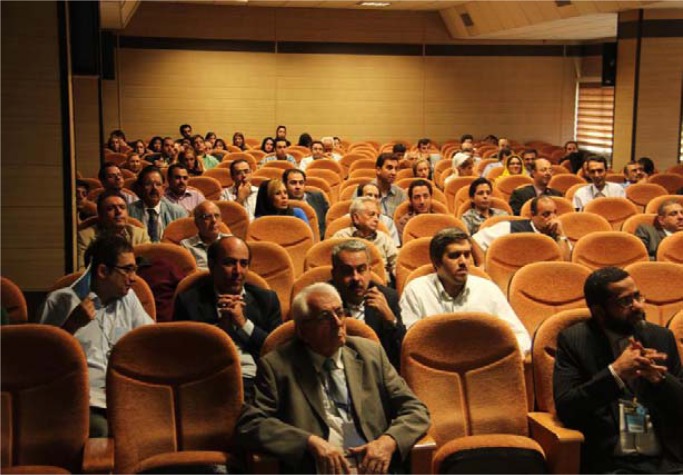
Audiences at the main hall during live neurointervention

**Figure 7:**
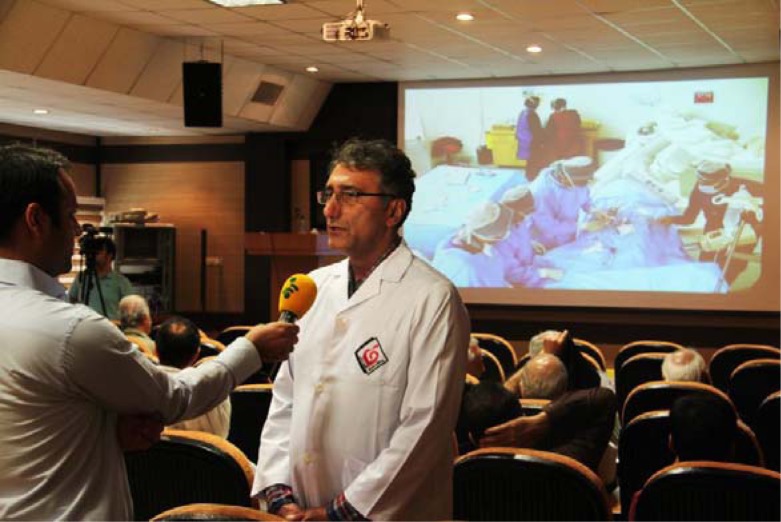
Dr. Askar Ghorbani, the Chairman of the First Iranian International Congress of Neurointervention

**Figure 8:**
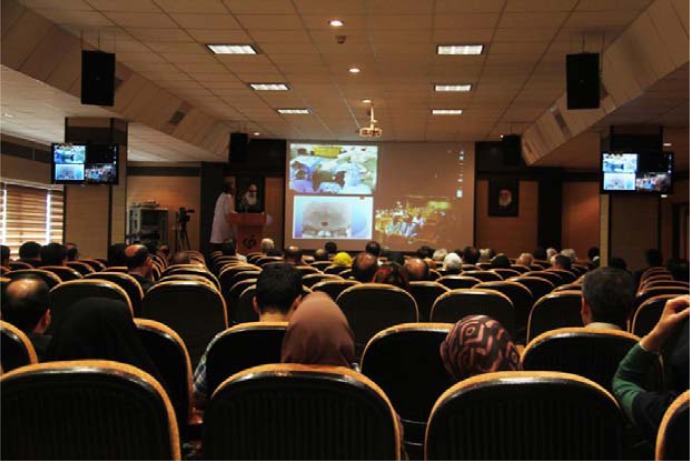
The main hall; audiences are watching live neurointervention

This two-day congress, which was coordinated by Dr. Askar Ghorbani and Dr. Faramarz Amiri (interventional neurologists), consisted of 5 sessions, each focusing on a common cerebrovascular disease and its treatment as below: stroke, subarachnoid hemorrhage and aneurysm, Galen venous malformations, arteriovenous malformations (AVM), and carotid artery; stenosis. It is noticeable that the neurointerventional procedures were conducted in catheterization laboratory and the audiences could watch the procedures lively and interactively communicate with the vascular specialists at catheterization laboratory (Figs. 2–8).

The main highlights of this event were as follows:

1. The live cases were shown directly from the catheterization laboratory where the interventionists could directly communicate with the guests and seek their opinions or answer their questions. Three challenging cases with aneurysms brought great excitement to the audience during the first day. Two patients with severe internal carotid artery stenosis underwent stenting and one AVM patient was treated by squid injection during the second day.
2. The panels, where the members were deeply engaged in debates about stroke and the endovascular procedures.

Close collaboration of different pertinent neurointerventional specialists including neurologists, cardiologists, neurosurgeons and radiologists.

In conclusion, it seems that this congress could assist a close cooperation between different neurointerventional specialists for better management of the patients suffering from cerebrovascular disorders. We hope that, in future, by the implementation of similar events and more widespread contribution of other countries especially in the Persian Gulf region, the main goal, that is more effective management of the patients, could be achieved.

